# Crystal structure and Hirshfeld surface analysis of 5-(3-nitro-1*H*-pyrazol-4-yl)-1*H*-tetra­zole

**DOI:** 10.1107/S2056989025007224

**Published:** 2025-08-21

**Authors:** Shannon E. Creegan, James A. Ridenour, Patrick A. Caruana, Ian D. Giles, Andrew T. Kerr

**Affiliations:** a US Naval Research Laboratory, Center for Biomolecular Science and Engineering, 4555 Overlook Ave., SW Washington, DC 20375, USA; b US Naval Research Laboratory Materials Chemistry and Dynamics Branch 4555 Overlook Ave SW Washington DC 20375 USA; Vienna University of Technology, Austria

**Keywords:** crystal structure, Huisgen reaction, pyrazole-substituted tetra­zoles

## Abstract

5-(3-Nitro-1*H*-pyrazol-4-yl)tetra­zole, C_4_H_3_N_7_O_2_, was synthesized from cyano­pyrazole *via* the Huisgen reaction. The asymmetric unit contains two mol­ecules, each displaying notable torsion between the pyrazole and tetra­zole systems. N—H⋯N hydrogen bonds and π-stacking inter­actions create a double-wide mol­ecular chain, while further N—H⋯N and weaker C—H⋯N inter­actions stitch these chains into a supra­molecular hydrogen-bonded framework. From a Hirshfeld surface analysis, the closest contacts between mol­ecules are through the N—H⋯N inter­actions between the tetra­zole and pyrazole rings with N⋯H/H⋯N making up the largest contributing contacts in the fingerprint plot.

## Abstract 2

5-(3-Nitro-1*H*-pyrazol-4-yl)tetra­zole crystallizes with two mol­ecules of nearly identical conformation in the asymmetric unit.

## 1. Chemical context

Heterocyclic systems are an area of inter­est due to their wide range of applications in energetic materials, pharmaceuticals, and dyes. These highly tailorable systems are useful for material characteristic modifications (*e.g.* solubility, polarity, density, *etc*.) and can be found in many natural products. As part of ongoing research, 5-(3-nitro-1*H*-pyrazol-4-yl)tetra­zole was isolated following literature procedures (Shkineva *et al.*, 2022 ▸) utilizing the Huisgen reaction for the formation of pyrazole-substituted tetra­zoles. This application of the Huisgen reaction occurs under standard conditions, refluxing 1,3-dipolar tri­ethyl­ammonium azide with a cyano­pyrazole to synthesize a tetra­zole.
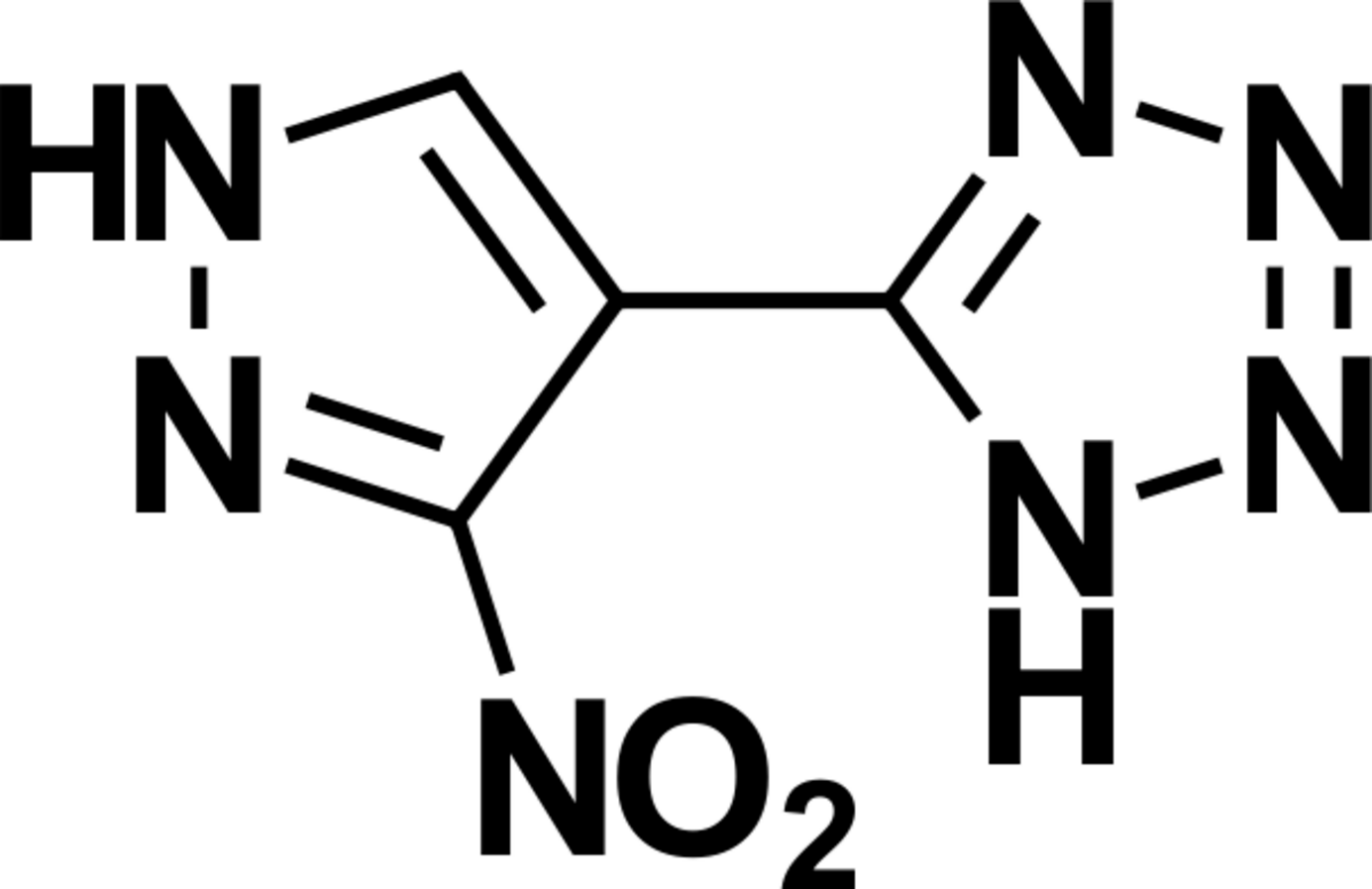


## 2. Structural commentary

**Figure 1:**
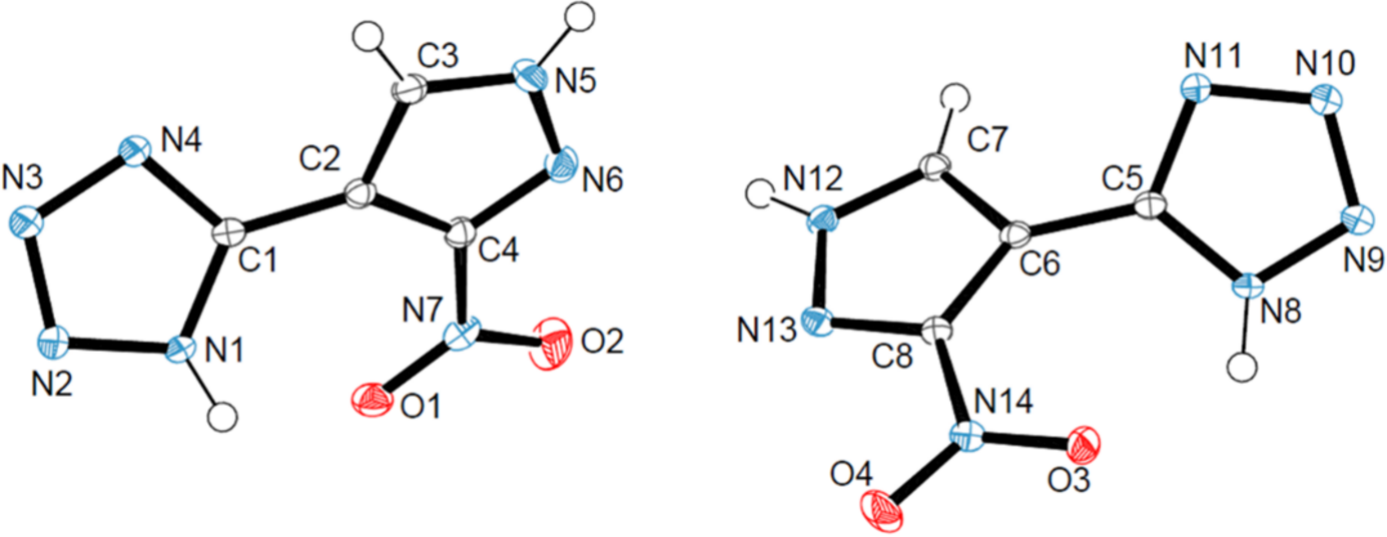
The two mol­ecules [1 (C1 C4) and 2 (C5–C8)] in the asymmetric unit shown with displacement ellipsoids at the 50% probability level; H atoms are given as spheres of arbitrary radius.

**Figure 2:**
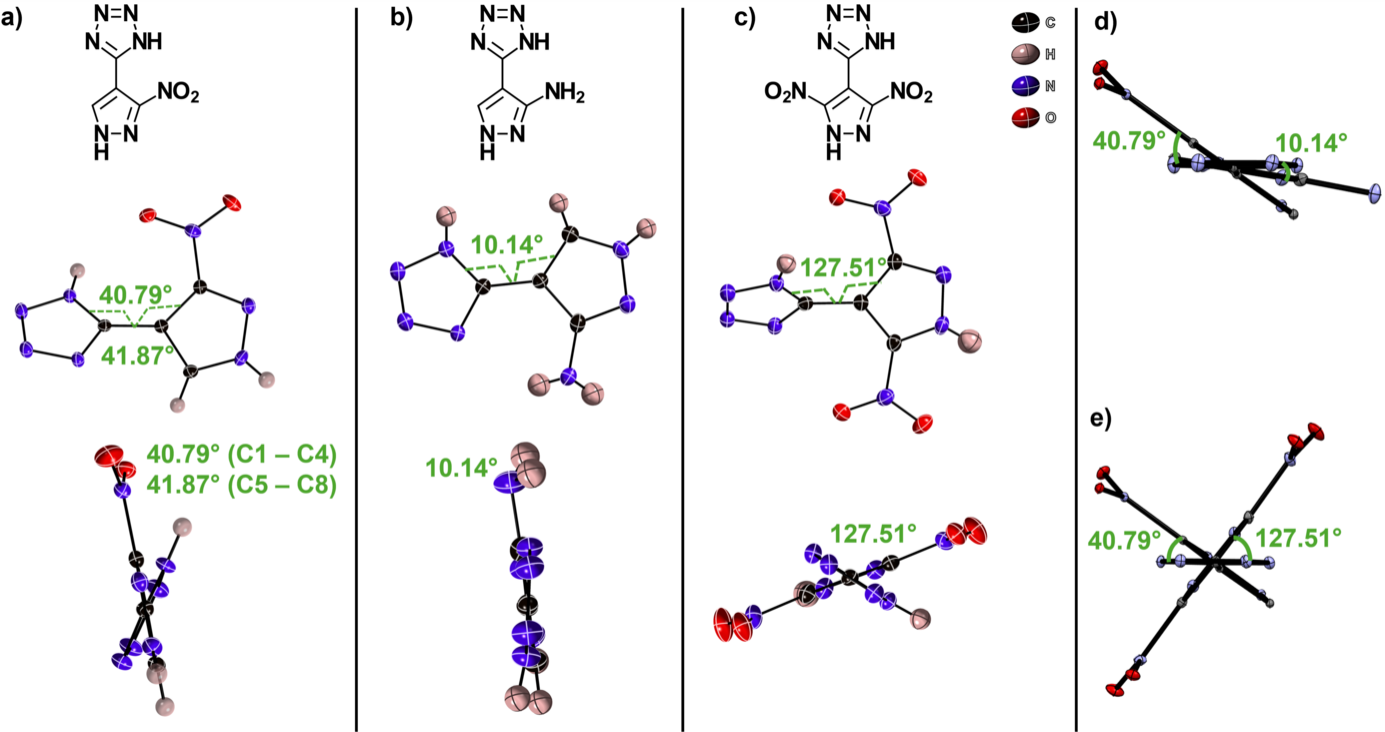
Mol­ecular structures of (*a*) 5-(3-nitro-1*H*-pyrazol-4-yl)tetra­zole and (*b*,*c*) of similar systems with torsion angles indicated; displacement ellipsoids are drawn at the 50% probability level. Structural overlay of the title compound with the amino (*d*) and di­nitro (*e*) systems for comparison; displacement ellipsoids are drawn at the 10% probability level.

The title compound crystallizes in the ortho­rhom­bic Sohnke space group *P*2_1_2_1_2_1_ with the asymmetric unit containing two mol­ecules of 5-(3-nitro-1*H*-pyrazol-4-yl)tetra­zole (Fig. 1 ▸; mol­ecule 1: C1–C4, and mol­ecule 2: C5–C8). All bond lengths are within expected range when compared to similar pyrazole/tetra­zole systems (see section 5). The tetra­zole and pyrazole rings are independently planar but non-planar to each other with N—C—C—C torsion angles of 40.8 (3)° in mol­ecule 1 (N1—C1—C2—C4) and 41.9 (3)° in mol­ecule 2 (N8—C5—C6—C8) (Fig. 2 ▸*a*). The non-planarity of each of the mol­ecules is likely driven by steric hindrance from the nitro group, which is seen in other reported pyrazole-tetra­zole mol­ecules (Hanghong *et al.*, 2024 ▸; Kumbar *et al.*, 2018 ▸), and/or influenced by the dominant N—H⋯N supra­molecular inter­actions discussed below.

## 3. Supra­molecular features

**Figure 3:**
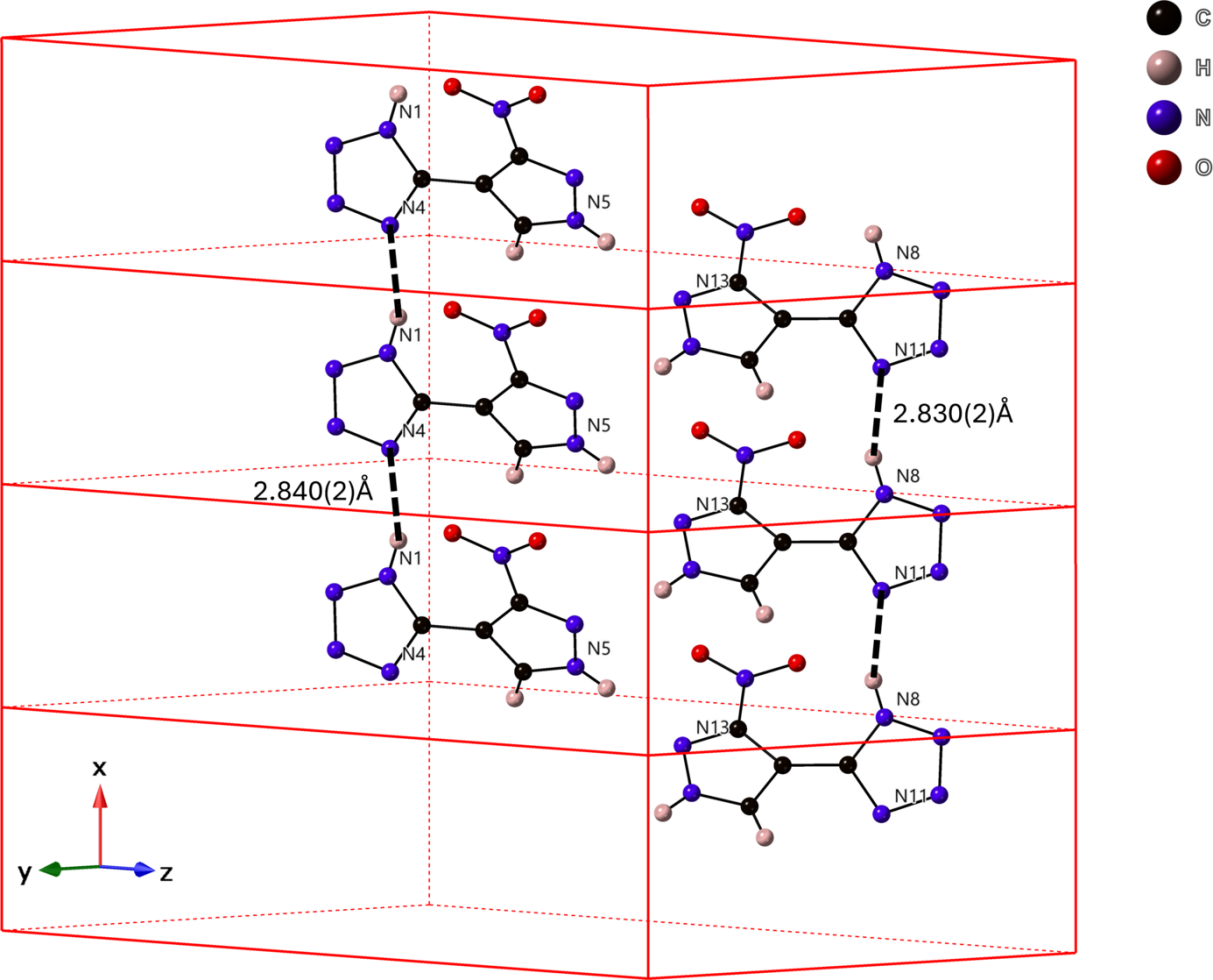
N—H⋯N hydrogen-bonding inter­actions (dotted lines) between symmetry-equivalent mol­ecules (N1⋯N4 in mol­ecule 1; N8⋯N11 in mol­ecule 2) generating chains.

**Figure 4:**
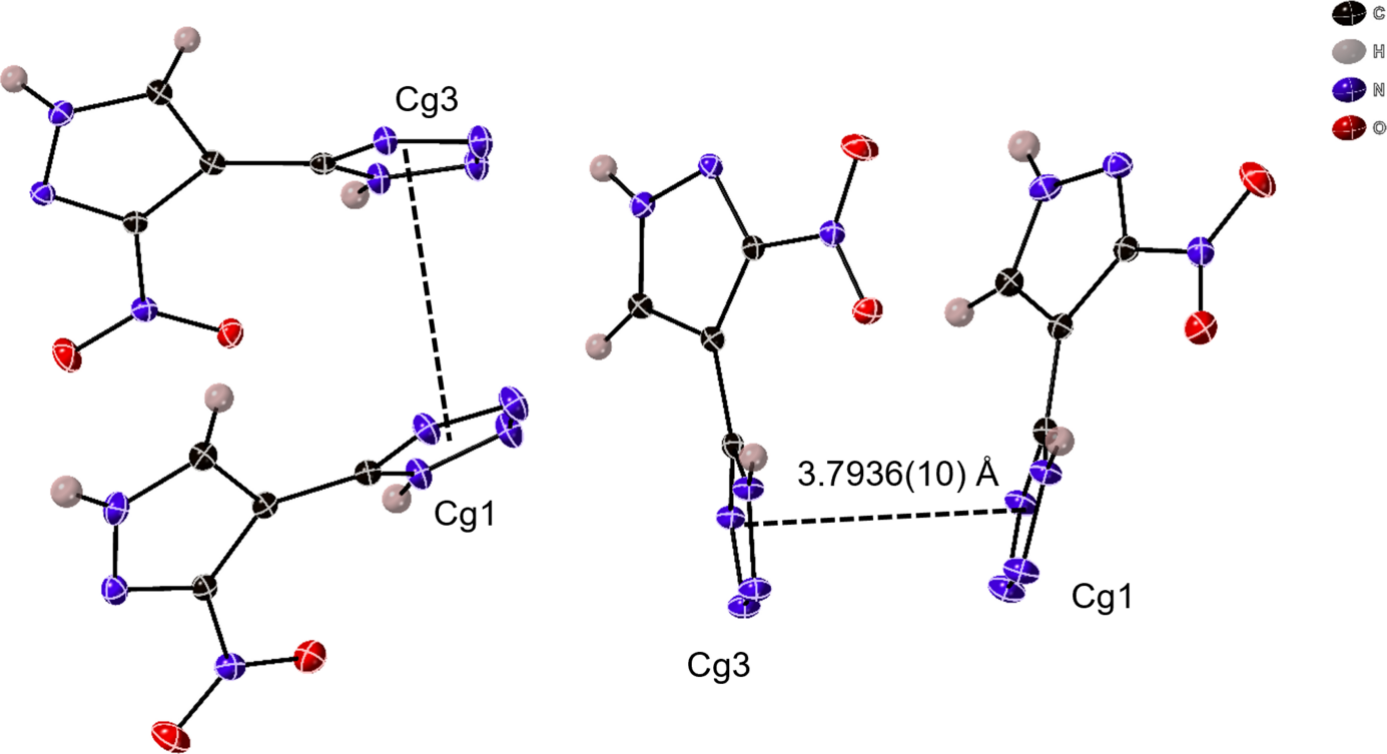
Mol­ecules 1 and 2 are connected *via* π–π stacking inter­actions between *Cg*1 (containing N3) and *Cg*3 (containing N10).

**Figure 5:**
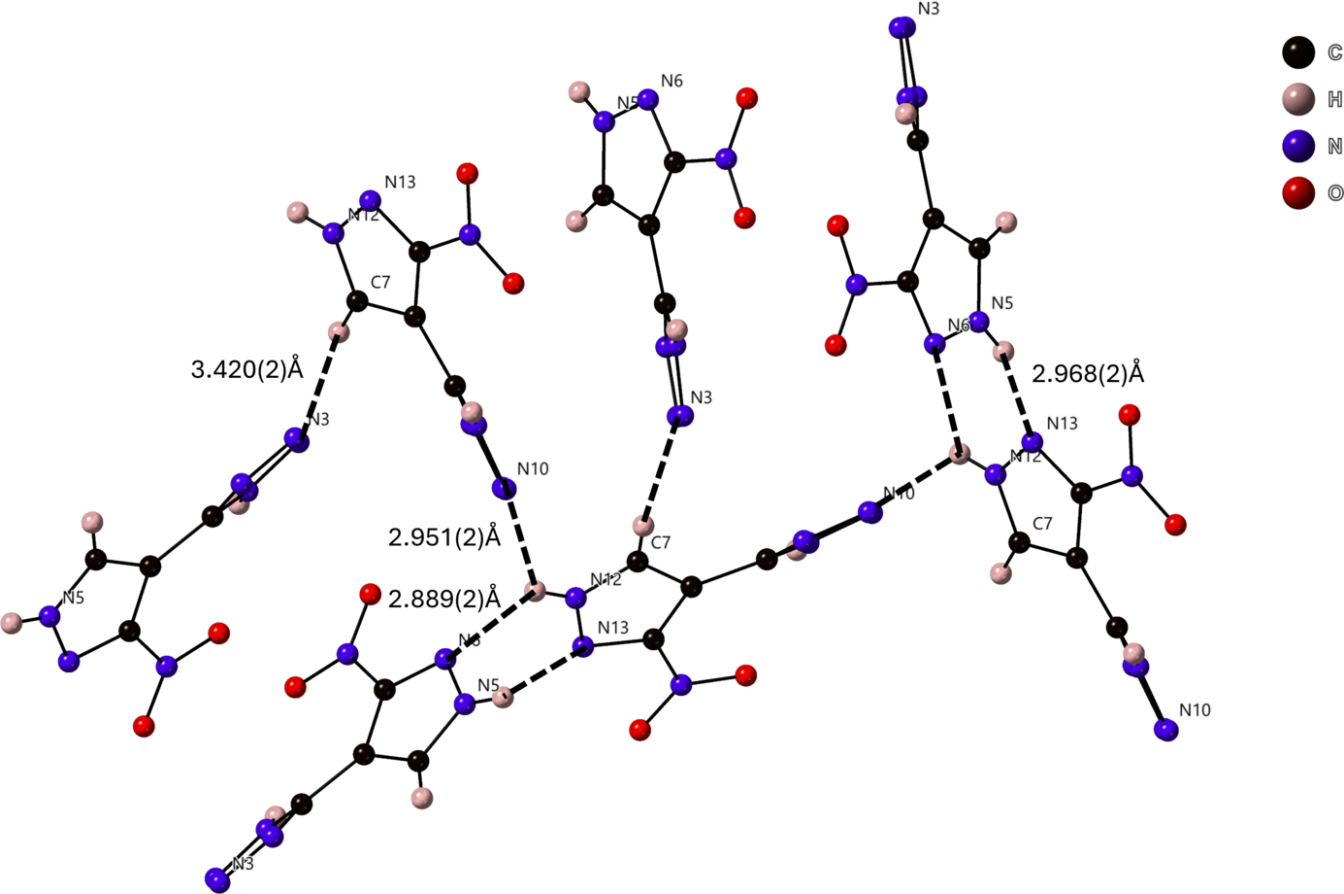
N—H⋯N hydrogen-bonding inter­actions between N5⋯N13, N12⋯N6 and N12⋯N10 as well as C—H⋯N inter­actions between C7⋯N3 weave the chains shown in Fig. 4 ▸ into a supra­molecular framework.

The supra­molecular packing inter­actions observed are primarily N—H⋯N and weaker C—H⋯N hydrogen-bonding inter­actions (Table 1 ▸). Each individual symmetrically equivalent mol­ecule is hydrogen-bound together through N—H⋯N inter­actions, at N⋯N distances of 2.840 (2) Å for N1⋯N4 and 2.830 (2) Å for N8⋯N11, to make chains of mol­ecule 1 and mol­ecule 2 parallel to [100] (Fig. 3 ▸). Further, the tetra­zole rings of mol­ecules 1 and 2 are π-stacking at a *Cg*⋯*Cg* distance of 3.7936 (10) Å with a *β* angle of 19.5°, *Cg*_perp_ of 3.7178 (7) Å (Janiak, 2000 ▸), and a slippage of 1.269 Å to form a double-wide chain (Fig. 4 ▸). These chains further inter­act with one another to make a supra­molecular framework *via* a bifurcated N—H⋯N hydrogen bond [N12⋯N6 = 2.889 (2) Å, N12⋯N10 = 2.950 (2) Å], a single N—H⋯N inter­action [N5⋯N13 = 2.968 (2) Å], and a C—H⋯N inter­action [C7⋯N3 = 3.420 (2) Å], as shown in Fig. 5 ▸.

## 4. Hirshfeld surface analysis

**Figure 6:**
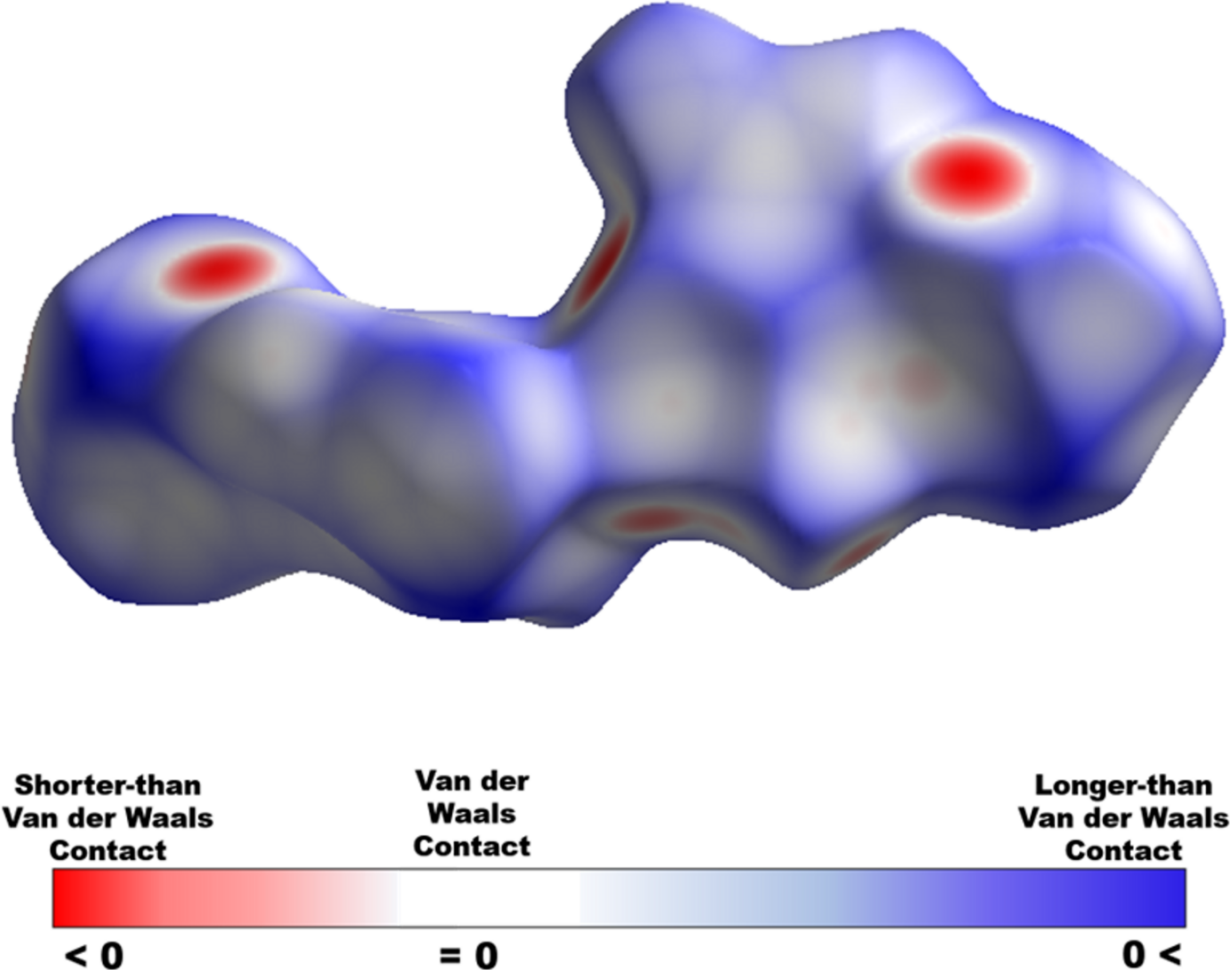
Hirshfeld surface displayed for the asymmetric unit. The region’s color indicates if a contact distance is shorter than (red), equal to (white), or longer than (blue) the van der Waals separation.

**Figure 7:**
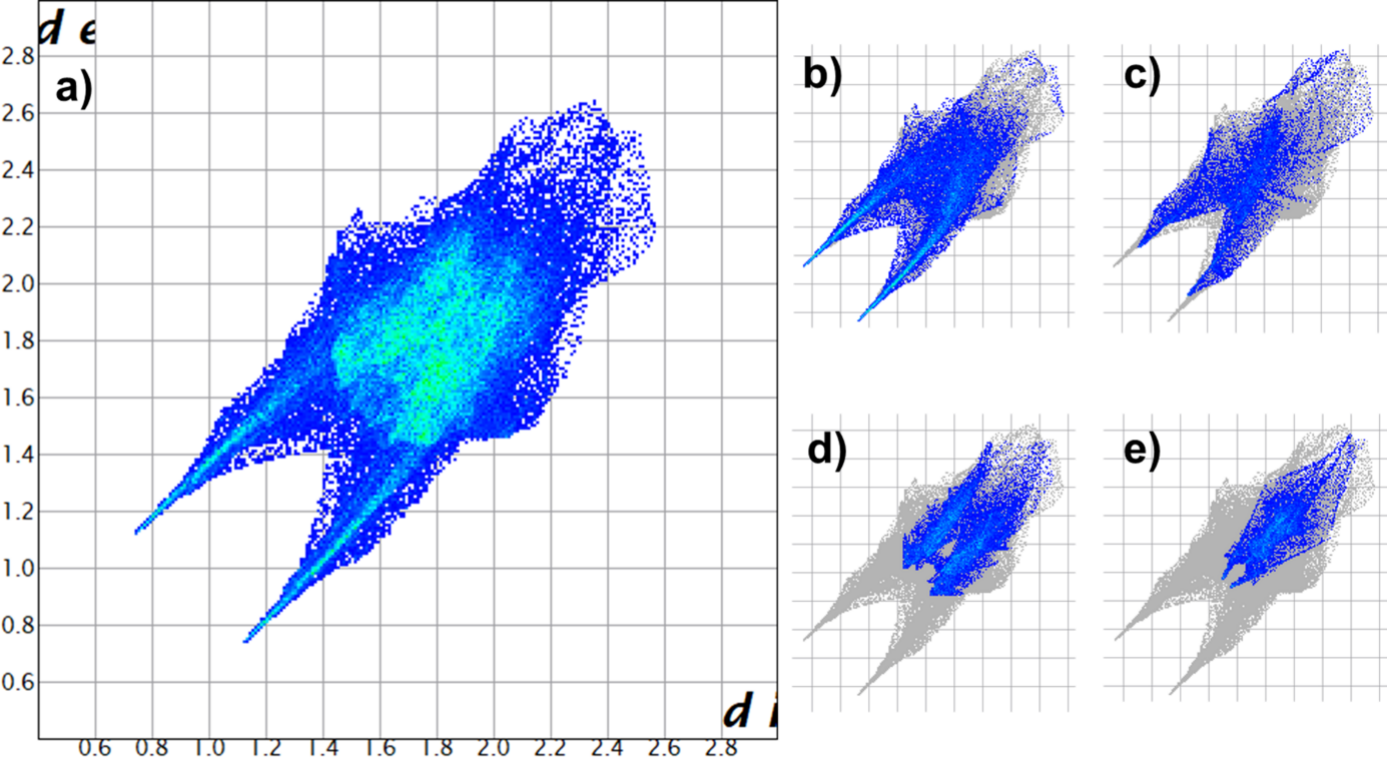
Fingerprint plots for the asymmetric unit of inter­actions greater than 10%; (*a*) all inter­actions, (*b*) N⋯H/H⋯N, (*c*) O⋯H/H⋯O, (*d*) N⋯O/O⋯N, (*e*) N⋯N inter­actions.

The Hirshfeld surface (Fig. 6 ▸) and the associated two-dimensional fingerprint plots (Fig. 7 ▸) of the crystal structure were generated over *d*_norm_ using *CrystalExplorer* (Spackman *et al.*, 2021 ▸). The areas of closest contact are associated with N—H⋯N inter­actions between the tetra­zole and pyrazole rings, seen as red areas in Fig. 6 ▸. From the fingerprint plots, N⋯H/H⋯N (31.5%) and N⋯O/O⋯N (18.4%) (Fig. 7 ▸*b*,*d*) contacts are the largest overall contributors, found in the lighter blue region of the fingerprint plot, with O⋯H/H⋯O (13.4%) (Fig. 7 ▸*c*) and N⋯N (10.9%) (Fig. 7 ▸*e*) contacts being midlevel contributors. Smaller contributions, less than 10%, are made by C⋯O/O⋯C (8.4%), N⋯C/C⋯N (6.7%), C⋯H/H⋯C (4.8%), O⋯O (3.4%) and H⋯H (2.3%) with the contribution of C⋯C contacts being less than 1%.

## 5. Database survey

A search of the Cambridge Structural Database (CSD, version 5.46, update November 2024; Groom *et al.*, 2016 ▸) yielded twenty-four entries containing 5-(3-nitro-1*H*-pyrazol-4-yl)tetra­zole as either a backbone structure or metal coordinating ligand. The most similar structures are 3-amino-4-tetra­zole-pyrazole (ENAGAE; Deng *et al.*, 2019 ▸) and 3,5-di­nitro­pyrazolyl-tetra­zole (VUSRUZ; Benz *et al.*, 2020 ▸). The pyrazole and tetra­zole rings exhibit the smallest torsion angle in the 3-amino structure, with a torsion angle of 10.14° (N1—C3—C1—C4) (Fig. 2 ▸*b*), while the di­nitro group displays the most torsion, 126.51° (N8—C4—C2—C3) (Fig. 2 ▸*c*). The torsion angles are graphically compared to the title compound using *Mercury* (Macrae *et al.*, 2020 ▸) structure overlay plots in Fig. 2 ▸*d* and 2*e*.

## 6. Synthesis and crystallization

5-(3-Nitro-1*H*-pyrazol-4-yl)tetra­zole was synthesized according to a literature procedure (Shkineva *et al.*, 2022 ▸). A mixture of cyano­pyrazole (0.497 g, 3.60 mmol), sodium azide (0.307 g, 4.72 mmol, 1.3 equiv.), tri­ethyl­amine hydro­chloride (0.6514 g, 4.73 mmol, 1.3 equiv.) and toluene (11 ml) was refluxed at 393 K for 16 h before cooling to ambient temperature. The resulting mixture was a clear, colorless liquid with yellow aggregates. Water (33 ml) was added to the mixture and stirred until all solids dissolved. The organic layer was removed and the aqueous layer acidified by the dropwise addition of hydro­chloric acid until the pH was between 0 and 1. The solution turned a lighter shade of yellow without immediate precipitation and, after twenty minutes, the solution was extracted with ethyl acetate (3 × 50 ml). The solvent was dried over sodium sulfate and concentrated *in vacuo* resulting in a viscous yellow oil. Minimal ethyl acetate was added, and the mixture was chilled in an ice bath forming a precipitate that was isolated by filtration and washed with cold ethyl acetate (0.311 g; as a 94.5:5.5 mixture of product to starting material based by NMR, 45%). Slow evaporation of the filtrate yielded 0.147 g of additional material that contained a mixture of 20% product and 80% starting material. The single crystal used for analysis was obtained *via* slow evaporation from ethanol. ^1^H NMR, (DMSO-*d_6_*) δ: 14.59 (*br.s*, 1 H, NH); 8.64 (*s*, 1 H, CH).

## 7. Refinement

Crystal data, data collection, and structure refinement details are summarized in Table 2 ▸. All hydrogen atoms on carbon and nitro­gen atoms were placed at their idealized positions and allowed to ride on the coordinates of their parent atoms [*U*_iso_(H) fixed at 1.2*U*_eq_(C,N)].

## Supplementary Material

Crystal structure: contains datablock(s) I. DOI: 10.1107/S2056989025007224/wm5766sup1.cif

Structure factors: contains datablock(s) I. DOI: 10.1107/S2056989025007224/wm5766Isup5.hkl

Cover Letter. DOI: 10.1107/S2056989025007224/wm5766sup3.docx

We made a mistake in the cif and corrected it but can't replace the original. DOI: 10.1107/S2056989025007224/wm5766sup4.txt

CCDC reference: 2480396

Additional supporting information:  crystallographic information; 3D view; checkCIF report

## Figures and Tables

**Table 1:** Hydrogen-bond geometry (Å, °)

*D*—H⋯*A*	*D*—H	H⋯*A*	*D*⋯*A*	*D*—H⋯*A*
C7—H7⋯N3^i^	0.93	2.51	3.420 (2)	167
N1—H1⋯N4^ii^	0.86	2.06	2.840 (2)	151
N5—H5⋯N13^iii^	0.86	2.14	2.968 (2)	162
N8—H8⋯N11^ii^	0.86	2.02	2.830 (2)	157
N12—H12⋯N6	0.86	2.20	2.889 (2)	137
N12—H12⋯N10^iv^	0.86	2.35	2.950 (2)	127

Symmetry codes: (i) 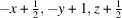; (ii) 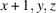; (iii) 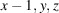; (iv) 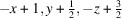.

**Table 2:** Experimental details

Crystal data	
Chemical formula	C_4_H_3_N_7_O_2_
*M*_r_	181.13
Crystal system, space group	Orthorhombic, *P*2_1_2_1_2_1_
Temperature (K)	100
*a*, *b*, *c* (Å)	4.9818 (1), 12.8064 (3), 21.5978 (5)
*V* (Å^3^)	1377.92 (5)
*Z*	8
Radiation type	Cu *K*α
μ (mm^−1^)	1.27
Crystal size (mm)	0.31 × 0.04 × 0.04

Data collection	
Diffractometer	Bruker Photon II CCD
Absorption correction	Multi-scan (*SADABS*; Krause *et al.*, 2015 ▸)
*T*_min_, *T*_max_	0.685, 0.754
No. of measured, independent and observed [*I* > 2σ(*I*)] reflections	14168, 2819, 2749
*R*_int_	0.028
(sin θ/λ)_max_ (Å^−1^)	0.625

Refinement	
*R*[*F*^2^ > 2σ(*F*^2^)], *wR*(*F*^2^), *S*	0.025, 0.061, 1.07
No. of reflections	2819
No. of parameters	235
H-atom treatment	H-atom parameters constrained
Δρ_max_, Δρ_min_ (e Å^−3^)	0.24, −0.22
Absolute structure	Flack *x* determined using 1109 quotients [(*I*^+^)−(*I*^−^)]/[(*I*^+^)+(*I*^−^)] (Parsons *et al.*, 2013 ▸)
Absolute structure parameter	0.00 (6)

Computer programs: *APEX3* and *SAINT* (Bruker, 2019 ▸), *SHELXT* (Sheldrick, 2015*a* ▸), *SHELXL* (Sheldrick, 2015*b* ▸), *SHELXTL* (Sheldrick, 2008 ▸) and *publCIF* (Westrip, 2010 ▸).

## supplementary crystallographic information

### 5-(3-Nitro-1H-pyrazol-4-yl)-1H-tetrazole Crystal data

**Table:**

C_4_H_3_N_7_O_2_	*D*_x_ = 1.746 Mg m^−^^3^
*M**_r_* = 181.13	Cu *K*α radiation, λ = 1.54178 Å
Orthorhombic, *P*2_1_2_1_2_1_	Cell parameters from 9948 reflections
*a* = 4.9818 (1) Å	θ = 4.0–74.6°
*b* = 12.8064 (3) Å	µ = 1.27 mm^−^^1^
*c* = 21.5978 (5) Å	*T* = 100 K
*V* = 1377.92 (5) Å^3^	Rod, colorless
*Z* = 8	0.31 × 0.04 × 0.04 mm
*F*(000) = 736	

### 5-(3-Nitro-1H-pyrazol-4-yl)-1H-tetrazole Data collection

**Table:**

Bruker Photon II CCD diffractometer	2819 independent reflections
Radiation source: 1uS microfocus	2749 reflections with *I* > 2σ(*I*)
Montel multilayer optics monochromator	*R*_int_ = 0.028
ω scans	θ_max_ = 74.5°, θ_min_ = 4.0°
Absorption correction: multi-scan (SADABS; Krause *et al.*, 2015)	*h* = −5→6
*T*_min_ = 0.685, *T*_max_ = 0.754	*k* = −14→16
14168 measured reflections	*l* = −22→27

### 5-(3-Nitro-1H-pyrazol-4-yl)-1H-tetrazole Refinement

**Table:**

Refinement on *F*^2^	Hydrogen site location: inferred from neighbouring sites
Least-squares matrix: full	H-atom parameters constrained
*R*[*F*^2^ > 2σ(*F*^2^)] = 0.025	*w* = 1/[σ^2^(*F*_o_^2^) + (0.0325*P*)^2^ + 0.3409*P*] where *P* = (*F*_o_^2^ + 2*F*_c_^2^)/3
*wR*(*F*^2^) = 0.061	(Δ/σ)_max_ < 0.001
*S* = 1.07	Δρ_max_ = 0.24 e Å^−^^3^
2819 reflections	Δρ_min_ = −0.22 e Å^−^^3^
235 parameters	Absolute structure: Flack *x* determined using 1109 quotients [(*I*^+^)-(*I*^-^)]/[(*I*^+^)+(*I*^-^)] (Parsons *et al.*, 2013)
0 restraints	Absolute structure parameter: 0.00 (6)
Primary atom site location: dual	

### 5-(3-Nitro-1H-pyrazol-4-yl)-1H-tetrazole Special details

**Table:**

Experimental. The hydrogen peak determination was made following the experimental results published by Shkineva *et al.* of a broad singlet NH peak at 14.59 accounting for a single hydrogen and the pyrazole CH peak at 8.64 as a singlet inDMSO-d6. The lack of a second NH signal was not unexpected and can be mostly likely attributed to rapid exchange on the tetrazole ring. The tetrazole NH peak is usually a very broad singlet in the 15-16 ppm region and is difficult to separate from the baseline at lower concertation.
Geometry. All esds (except the esd in the dihedral angle between two l.s. planes) are estimated using the full covariance matrix. The cell esds are taken into account individually in the estimation of esds in distances, angles and torsion angles; correlations between esds in cell parameters are only used when they are defined by crystal symmetry. An approximate (isotropic) treatment of cell esds is used for estimating esds involving l.s. planes.

### 5-(3-Nitro-1H-pyrazol-4-yl)-1H-tetrazole Fractional atomic coordinates and isotropic or equivalent isotropic displacement parameters (Å^2^)

**Table:**

	*x*	*y*	*z*	*U*_iso_*/*U*_eq_	
C1	0.4100 (3)	0.62085 (13)	0.39918 (8)	0.0115 (3)	
C2	0.3934 (3)	0.56191 (13)	0.45654 (8)	0.0118 (3)	
C3	0.2011 (3)	0.48620 (13)	0.46685 (8)	0.0136 (3)	
H3	0.073301	0.463185	0.438575	0.016*	
C4	0.5297 (3)	0.56563 (14)	0.51357 (8)	0.0122 (3)	
C5	0.7970 (3)	0.11907 (12)	0.72649 (7)	0.0104 (3)	
C6	0.7960 (3)	0.20638 (13)	0.68310 (7)	0.0102 (3)	
C7	0.6228 (3)	0.29043 (13)	0.68753 (8)	0.0109 (3)	
H7	0.490779	0.300077	0.717445	0.013*	
C8	0.9502 (3)	0.23144 (13)	0.63073 (8)	0.0105 (3)	
N1	0.6270 (3)	0.65514 (11)	0.36902 (7)	0.0131 (3)	
H1	0.790003	0.649609	0.381749	0.016*	
N2	0.5496 (3)	0.69956 (13)	0.31571 (7)	0.0163 (3)	
N3	0.2900 (3)	0.69265 (13)	0.31368 (7)	0.0172 (3)	
N4	0.1961 (3)	0.64375 (12)	0.36525 (6)	0.0146 (3)	
N5	0.2322 (3)	0.45188 (11)	0.52513 (7)	0.0158 (3)	
H5	0.133196	0.404306	0.541515	0.019*	
N6	0.4337 (3)	0.49950 (12)	0.55518 (7)	0.0151 (3)	
N7	0.7521 (3)	0.63238 (12)	0.53093 (7)	0.0152 (3)	
N8	1.0099 (3)	0.07153 (12)	0.75156 (7)	0.0123 (3)	
H8	1.174939	0.083842	0.742439	0.015*	
N9	0.9238 (3)	0.00135 (12)	0.79336 (7)	0.0145 (3)	
N10	0.6635 (3)	0.00620 (12)	0.79306 (7)	0.0143 (3)	
N11	0.5776 (3)	0.07882 (11)	0.75166 (6)	0.0121 (3)	
N12	0.6802 (3)	0.35523 (11)	0.64088 (6)	0.0118 (3)	
H12	0.596336	0.413000	0.634707	0.014*	
N13	0.8801 (3)	0.32180 (11)	0.60472 (7)	0.0117 (3)	
N14	1.1636 (3)	0.17244 (11)	0.60266 (7)	0.0124 (3)	
O1	0.8497 (3)	0.68602 (10)	0.48967 (6)	0.0182 (3)	
O2	0.8280 (3)	0.63091 (12)	0.58474 (6)	0.0276 (3)	
O3	1.2290 (2)	0.09068 (9)	0.62874 (6)	0.0154 (3)	
O4	1.2686 (3)	0.20548 (11)	0.55516 (6)	0.0230 (3)	

### 5-(3-Nitro-1H-pyrazol-4-yl)-1H-tetrazole Atomic displacement parameters (Å^2^)

**Table:**

	*U*^11^	*U*^22^	*U*^33^	*U*^12^	*U*^13^	*U*^23^
C1	0.0093 (7)	0.0124 (8)	0.0129 (8)	−0.0002 (6)	0.0002 (6)	−0.0010 (6)
C2	0.0099 (7)	0.0118 (8)	0.0137 (8)	0.0017 (6)	0.0005 (6)	−0.0005 (6)
C3	0.0100 (7)	0.0143 (8)	0.0164 (8)	−0.0001 (7)	−0.0004 (6)	0.0001 (7)
C4	0.0116 (7)	0.0121 (8)	0.0130 (8)	0.0019 (6)	0.0006 (6)	−0.0002 (6)
C5	0.0092 (7)	0.0110 (7)	0.0109 (7)	0.0000 (6)	0.0005 (6)	−0.0016 (6)
C6	0.0078 (6)	0.0125 (7)	0.0103 (7)	−0.0009 (6)	−0.0015 (6)	−0.0009 (6)
C7	0.0088 (7)	0.0131 (8)	0.0108 (8)	−0.0009 (6)	−0.0007 (6)	−0.0005 (6)
C8	0.0097 (7)	0.0111 (8)	0.0107 (8)	−0.0001 (6)	0.0002 (6)	−0.0009 (6)
N1	0.0077 (6)	0.0187 (7)	0.0131 (7)	0.0003 (5)	−0.0010 (5)	0.0033 (6)
N2	0.0123 (7)	0.0232 (8)	0.0134 (7)	0.0006 (6)	−0.0004 (6)	0.0054 (6)
N3	0.0117 (6)	0.0260 (8)	0.0139 (7)	−0.0002 (6)	−0.0006 (6)	0.0049 (6)
N4	0.0103 (6)	0.0212 (7)	0.0124 (7)	0.0000 (6)	−0.0004 (6)	0.0034 (6)
N5	0.0145 (7)	0.0145 (7)	0.0185 (7)	−0.0018 (6)	0.0026 (6)	0.0040 (6)
N6	0.0157 (7)	0.0152 (7)	0.0146 (7)	0.0030 (6)	0.0005 (5)	0.0014 (6)
N7	0.0125 (7)	0.0160 (7)	0.0170 (7)	0.0024 (6)	−0.0028 (6)	−0.0027 (6)
N8	0.0071 (6)	0.0155 (7)	0.0143 (7)	−0.0008 (5)	0.0008 (5)	0.0037 (6)
N9	0.0109 (7)	0.0163 (7)	0.0162 (7)	−0.0004 (6)	0.0012 (5)	0.0048 (6)
N10	0.0109 (7)	0.0162 (7)	0.0157 (7)	−0.0007 (6)	0.0004 (5)	0.0044 (6)
N11	0.0099 (6)	0.0138 (7)	0.0125 (7)	0.0002 (6)	0.0003 (5)	0.0024 (6)
N12	0.0103 (6)	0.0112 (6)	0.0138 (7)	0.0016 (5)	−0.0001 (5)	0.0000 (5)
N13	0.0116 (6)	0.0122 (7)	0.0113 (7)	−0.0004 (5)	0.0020 (5)	−0.0006 (6)
N14	0.0110 (7)	0.0130 (7)	0.0132 (7)	−0.0007 (5)	0.0012 (6)	−0.0008 (6)
O1	0.0135 (6)	0.0187 (6)	0.0225 (6)	−0.0032 (5)	−0.0002 (5)	0.0005 (5)
O2	0.0311 (8)	0.0337 (8)	0.0180 (7)	−0.0037 (7)	−0.0113 (6)	−0.0017 (6)
O3	0.0154 (6)	0.0140 (6)	0.0168 (6)	0.0044 (5)	0.0020 (5)	0.0027 (5)
O4	0.0250 (7)	0.0246 (7)	0.0195 (6)	0.0067 (6)	0.0142 (6)	0.0064 (5)

### 5-(3-Nitro-1H-pyrazol-4-yl)-1H-tetrazole Geometric parameters (Å, º)

**Table:**

C1—N4	1.326 (2)	C8—N14	1.438 (2)
C1—N1	1.336 (2)	N1—N2	1.341 (2)
C1—C2	1.453 (2)	N1—H1	0.8600
C2—C3	1.381 (2)	N2—N3	1.297 (2)
C2—C4	1.407 (2)	N3—N4	1.361 (2)
C3—N5	1.342 (2)	N5—N6	1.342 (2)
C3—H3	0.9300	N5—H5	0.8600
C4—N6	1.324 (2)	N7—O2	1.222 (2)
C4—N7	1.449 (2)	N7—O1	1.226 (2)
C5—N11	1.325 (2)	N8—N9	1.344 (2)
C5—N8	1.337 (2)	N8—H8	0.8600
C5—C6	1.459 (2)	N9—N10	1.298 (2)
C6—C7	1.383 (2)	N10—N11	1.359 (2)
C6—C8	1.404 (2)	N12—N13	1.336 (2)
C7—N12	1.336 (2)	N12—H12	0.8600
C7—H7	0.9300	N14—O4	1.2269 (19)
C8—N13	1.333 (2)	N14—O3	1.2329 (19)
			
N4—C1—N1	107.95 (14)	C1—N1—H1	125.5
N4—C1—C2	122.69 (15)	N2—N1—H1	125.5
N1—C1—C2	129.23 (15)	N3—N2—N1	106.67 (14)
C3—C2—C4	102.55 (15)	N2—N3—N4	110.29 (14)
C3—C2—C1	122.81 (16)	C1—N4—N3	106.11 (14)
C4—C2—C1	134.51 (16)	N6—N5—C3	113.03 (14)
N5—C3—C2	107.52 (15)	N6—N5—H5	123.5
N5—C3—H3	126.2	C3—N5—H5	123.5
C2—C3—H3	126.2	C4—N6—N5	103.44 (14)
N6—C4—C2	113.46 (15)	O2—N7—O1	125.24 (16)
N6—C4—N7	118.54 (15)	O2—N7—C4	118.27 (15)
C2—C4—N7	127.99 (16)	O1—N7—C4	116.49 (14)
N11—C5—N8	108.11 (14)	C5—N8—N9	108.85 (13)
N11—C5—C6	123.96 (15)	C5—N8—H8	125.6
N8—C5—C6	127.73 (15)	N9—N8—H8	125.6
C7—C6—C8	102.66 (14)	N10—N9—N8	106.48 (14)
C7—C6—C5	123.67 (15)	N9—N10—N11	110.51 (15)
C8—C6—C5	133.67 (15)	C5—N11—N10	106.05 (14)
N12—C7—C6	107.31 (14)	N13—N12—C7	113.66 (14)
N12—C7—H7	126.3	N13—N12—H12	123.2
C6—C7—H7	126.3	C7—N12—H12	123.2
N13—C8—C6	113.23 (14)	C8—N13—N12	103.14 (13)
N13—C8—N14	118.17 (14)	O4—N14—O3	124.21 (14)
C6—C8—N14	128.59 (15)	O4—N14—C8	119.11 (14)
C1—N1—N2	108.98 (14)	O3—N14—C8	116.68 (14)
			
N4—C1—C2—C3	31.1 (3)	C2—C1—N4—N3	−176.02 (16)
N1—C1—C2—C3	−144.16 (19)	N2—N3—N4—C1	0.0 (2)
N4—C1—C2—C4	−144.0 (2)	C2—C3—N5—N6	0.0 (2)
N1—C1—C2—C4	40.8 (3)	C2—C4—N6—N5	0.00 (19)
C4—C2—C3—N5	−0.01 (18)	N7—C4—N6—N5	179.22 (14)
C1—C2—C3—N5	−176.40 (15)	C3—N5—N6—C4	0.00 (19)
C3—C2—C4—N6	0.0 (2)	N6—C4—N7—O2	−5.8 (2)
C1—C2—C4—N6	175.75 (18)	C2—C4—N7—O2	173.33 (17)
C3—C2—C4—N7	−179.12 (16)	N6—C4—N7—O1	174.02 (15)
C1—C2—C4—N7	−3.4 (3)	C2—C4—N7—O1	−6.9 (3)
N11—C5—C6—C7	37.2 (3)	N11—C5—N8—N9	−0.58 (18)
N8—C5—C6—C7	−137.05 (18)	C6—C5—N8—N9	174.39 (16)
N11—C5—C6—C8	−143.90 (18)	C5—N8—N9—N10	0.43 (19)
N8—C5—C6—C8	41.9 (3)	N8—N9—N10—N11	−0.1 (2)
C8—C6—C7—N12	0.15 (17)	N8—C5—N11—N10	0.50 (17)
C5—C6—C7—N12	179.35 (14)	C6—C5—N11—N10	−174.71 (15)
C7—C6—C8—N13	−0.28 (19)	N9—N10—N11—C5	−0.24 (19)
C5—C6—C8—N13	−179.36 (17)	C6—C7—N12—N13	0.02 (19)
C7—C6—C8—N14	−179.36 (16)	C6—C8—N13—N12	0.29 (18)
C5—C6—C8—N14	1.6 (3)	N14—C8—N13—N12	179.47 (14)
N4—C1—N1—N2	−0.21 (19)	C7—N12—N13—C8	−0.19 (18)
C2—C1—N1—N2	175.60 (17)	N13—C8—N14—O4	−1.9 (2)
C1—N1—N2—N3	0.2 (2)	C6—C8—N14—O4	177.14 (17)
N1—N2—N3—N4	−0.1 (2)	N13—C8—N14—O3	178.01 (14)
N1—C1—N4—N3	0.12 (19)	C6—C8—N14—O3	−3.0 (2)

### 5-(3-Nitro-1H-pyrazol-4-yl)-1H-tetrazole Hydrogen-bond geometry (Å, º)

**Table:**

*D*—H···*A*	*D*—H	H···*A*	*D*···*A*	*D*—H···*A*
C7—H7···N3^i^	0.93	2.51	3.420 (2)	167
N1—H1···N4^ii^	0.86	2.06	2.840 (2)	151
N5—H5···N13^iii^	0.86	2.14	2.968 (2)	162
N8—H8···N11^ii^	0.86	2.02	2.830 (2)	157
N12—H12···N6	0.86	2.20	2.889 (2)	137
N12—H12···N10^iv^	0.86	2.35	2.950 (2)	127

Symmetry codes: (i) −*x*+1/2, −*y*+1, *z*+1/2; (ii) *x*+1, *y*, *z*; (iii) *x*−1, *y*, *z*; (iv) −*x*+1, *y*+1/2, −*z*+3/2.
