# Supplementary material for: Crystal structure and Hirshfeld surface analysis of 5-(3-nitro-1H-pyrazol-4-yl)-1H-tetra­zole
Source: Acta Crystallogr E Crystallogr Commun. 2025 Aug 21;81(Pt 9):861–4. doi: 10.1107/S2056989025007224 (PMC12412694; doi:10.1107/S2056989025007224)
Supplement: Supplementary file 3 [file e-81-00861-sup3.docx]

Dr. Shannon E. Creegan

Center for Biomolecular Science and Engineering

U.S. Naval Research Laboratory

*Acta Crystallographica Section E, Crystallographic Communications*

2 Abbey Square, Chester, England, CH1 2HU

Dear Editor,

We wish to submit our original research paper “Crystal structure of 5-(3-nitro-1H-pyrazol-4-yl)tetrazole with Hirshfeld surface analysis” to be published by *Acta Crystallographica Section E.*

In this paper we describe the synthesis of the title compound and placed the compound within the context of other structurally similar materials. The comparison demonstrates that nitro groups play a role in not only the packing of molecules, but also the torsion angles between rings. This information provides valuable insight to the behaviors of heterocyclic systems as the wider research community explores the best ways to utilize these highly tailorable systems.

We believe this manuscript is appropriate for publication by Acta Cryst. E as it focuses on the structure of the compound and the effect its functional groups have on the torsion between the pyrazole and tetrazole systems.

This paper has not been published elsewhere and is not under consideration by another journal. All authors have approved the manuscript and agree with its submission to *Acta Crystallographica Section E.* The information covered reflects original work performed by the authors and we have no conflict of interests to disclose.

Thank you for your consideration.

Sincerely,

Dr. Shannon E. Creegan

Center for Biomolecular Science and Engineering

U.S. Naval Research Laboratory

4555 Overlook Ave., SW Washington, DC 20375

[shannon.e.creegan.civ@us.navy.mil](mailto:shannon.e.creegan.civ@us.navy.mil)

Co-authors

Dr. James A. Ridenour

Materials Chemistry and Dynamics Branch

U.S. Naval Research Laboratory

Dr. Patrick A. Caruana, Dr. Ian D. Giles, and Dr. Andrew T. Kerr

Center for Biomolecular Science and Engineering

U.S. Naval Research Laboratory
